# Breakup dynamics of weakly bound nuclei at energies around the Coulomb barrier

**DOI:** 10.1016/j.fmre.2023.10.006

**Published:** 2023-11-08

**Authors:** L. Yang, C.J. Lin, N.R. Ma, P.W. Wen, H.M. Jia, F. Yang

**Affiliations:** a China Institute of Atomic Energy, PO Box 275(10), Beijing 102413, China; b Department of Physics, Guangxi Normal University, Guilin 541004, China

**Keywords:** Breakup reaction, Reaction mechanism, Near-barrier energies, Stable weakly bound nuclei, Light unstable nuclei

## Abstract

The present work provides a literature survey of breakup reactions induced by weakly bound nuclei at energies around the Coulomb barrier. We review the inclusive and exclusive breakup data of stable weakly bound nuclei ${}^{6,7}$Li and ${}^{9}$Be, as well as light radioactive projectiles reported within the last decade. Several theoretical and data analysis tools used to describe the data are reviewed as well. Similarities and differences in the behavior of breakup reactions involving these weakly bound nuclei are discussed. It is found that, for ${}^{6,7}$Li and ${}^{9}$Be, transfer-triggered breakup is a significant mode, which, however, is not observed in drip-line nuclear systems. Moreover, differences in the breakup dynamics and the contribution of breakup to the total reaction cross section at energies close to the Coulomb barrier seem to emerge between neutron-halo and proton-halo systems. Possible explanations for the observed differences are discussed.

## 1 Introduction

It is known that, the average binding energy per nucleon is about 8 MeV for typical nuclei, resulting in a tightly bound nuclear structure. However, there exist exotic nuclei where the nucleons are weakly bound to each other. For instance, the binding energy of deuteron is 2.22 MeV, and separation energy of alpha in ${}^{6}$Li is around 1.47 MeV. Such nuclei are hence referred to as weakly bound nuclei, and their weakly bound nature is crucial for understanding nuclear structure [1], [2], nuclear potential [3] and consequently the reaction dynamics [4], [5]. It becomes more intriguing for nuclei located closest to the drip line, which are characterized by the extremely weakly bound nucleon/nucleons. Some of these drip line nuclei exhibit exotic halo structures [2], [6]. An important feature of collisions of weakly bound nuclei is the high probability of breakup reaction, leading to the formation of many-body open quantum systems (OQSs): The large environment provided by the breakup continuum could strongly interact with the subsystems, like, the elastic scattering channel and fusion reactions. Such coupling effect becomes particularly significant at energies near the Coulomb barrier. Therefore, understanding the breakup mechanism, especially in the energy region near the Coulomb barrier, is crucial for comprehending the reaction dynamics of weakly bound nuclear systems.

The study of the breakup process has been ongoing for several decades. In the first twenty years or so, the focus was the breakup reactions induced by deuteron (1950–1970) [7]. In the 1970s, stable heavy-ions started to be used as projectiles, thanks to the development of more powerful accelerators. From 1985 up to date, the nuclear territory has rapidly expanded due to the new generation of facilities and increased availability of unstable isotopes far from the valley of stability. As a result, reactions and structures of such unstable nuclei have become one of the major subjects in nuclear physics.

With the accumulation of breakup measurements at near-barrier energies, it is therefore timely to review the available data on both stable and unstable weakly bound nuclei, and to summarise what we can learn from these measurements and to provide indications for future research. In this review article, our focus is primarily on experimental studies of breakup reactions of stable weakly bound heavy-ions, namely, ${}^{6,7}$Li and ${}^{9}$Be. We also include studies on light (A<20) radioactive nuclei, since the present generation of accelerator facilities can produce reasonably intensive beams of such nuclei for experimental studies at the energies of interest.

The structure of the review is as follows. After the brief description of the breakup process and an introduction of the theoretical models used to describe the breakup data, we then give a survey of the available data of stable heavy-ions as well as neutron-rich and proton-rich unstable nuclei. Finally, we give our conclusions and future outlook of this topic.

## 2 Experimental and theoretical descriptions of breakup reaction

A breakup reaction occurs when a weakly bound nucleus is excited above its breakup threshold, either through the long-range Coulomb or short-range nuclear interactions. If the nucleus is populated to long-lived (narrow) resonances, the “asymptotic” breakup may occur on the outgoing trajectory, relatively far way from the target. Instead, a breakup from non-resonant continuum or broad resonant states called “prompt” breakup, occurs close to the target. The information about the projectile excitation and the location of breakup could be revealed by the relative energy $E_{\text{rel}}$ of the breakup fragments [8], which is defined as:

(1) $$E_{\text{rel}}=\frac{m_{1}E_{2}+m_{2}E_{1}-2\sqrt{m_{1}E_{1}m_{2}E_{2}}\cos\theta_{12}}{m_{1}+m_{2}}$$

where, $m_{1}$, $m_{2}$ and $E_{1}$, $E_{2}$ are the masses and energies of the breakup fragments and $\theta_{12}$ their opening angles. If breakup occurs asymptotically far from the target nucleus, the observed $E_{\text{rel}}$ will be expressed as the sum of the Q value of the breakup reaction and the excitation energy of the resonant state through which breakup occurs. However, if the breakup occurs via the short-lived states, it is expected to happen close to the target. In this case, the strong Coulomb and nuclear interactions between the breakup fragments and the target nucleus will alter the trajectories of the fragments, resulting in a distorted $E_{\text{rel}}$ distribution. Therefore, it is possible to separate the near-target breakup from the asymptotic breakup using the structure of $E_{\text{rel}}$ distribution.

**Fig. 1:**
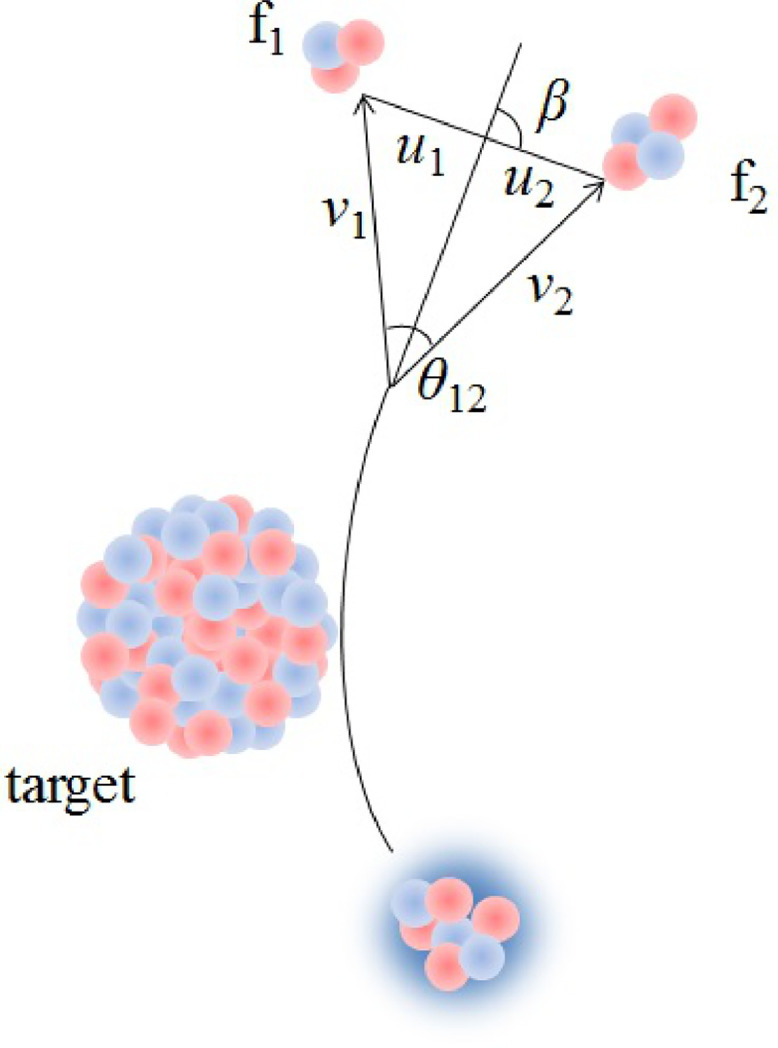
**Schematic illustration of a breakup process.**$v_{1}$, $v_{2}$ and $u_{1}$, $u_{2}$ are the velocities of breakup fragments (${\text{f}}_{1}$ and ${\text{f}}_{2}$) in the laboratory and composite rest frames, respectively. The laboratory frame opening angle is $\theta_{12}$. β represents the orientation of the relative velocity with respect to that of the center of mass.

The timescale of breakup can be further determined from the distributions of $\theta_{12}$ and β, which is the orientation of the relative momentum of the breakup fragments in their center-of-mass frame [9], [10], [11]. The demonstrations of $\theta_{12}$ and β can be seen in Fig. 1. For an asymptotic breakup, the corresponding velocities are depicted in Fig. 1 as well. The velocities of the fragments ${\text{f}}_{1}$ and ${\text{f}}_{2}$ in the lab frame are $v_{1}$ and $v_{2}$, which are deduced from the measured energies $E_{1}$ and $E_{2}$. The velocities of the fragments in the composite rest frame are $u_{1}$ and $u_{2}$, obtained from the conservation of momentum and the relative energy $E_{\text{rel}}\;=\;\mu (u_{1}\;+\;u_{2}$)/2, where μ stands for the reduced mass of the two fragments. For a given initial excitation energy, the relation between $\theta_{12}$ and β can be expressed as:

(2) $$\sin\beta =\frac{v_{1}v_{2}\sin\theta_{12}}{{\left(v_{2}^{2}u_{1}^{2}+v_{1}^{2}u_{2}^{2}+2u_{1}u_{2}v_{1}v_{2}\cos\theta_{12}\right)}^{1/2}}$$

However, for events from breakup close to the target, the correlation between β and $\theta_{12}$ becomes distorted due to the post-breakup Coulomb acceleration of the breakup fragments [9]. In this sense, this correlation can provide information about the location of breakup.

Consequently, to investigate the breakup mechanism of a weakly bound nuclear system, it is necessary to have complete kinematic information for each breakup fragment. This requires efficiently detecting the breakup fragments in coincidence. When all of the outgoing breakup fragments are recorded, it is referred to as an *exclusive* measurement. Otherwise, if one or more fragments are not detected in the experiment, it is considered an *inclusive* measurement with respect to the unobserved fragment(s).

From a theoretical perspective, various classical, semiclassical and quantum theories have been developed to describe the breakup reaction. Detailed discussions and equations related to these theoretical frameworks can be found in Refs. [7], [12], [13], [14], [15]. In this paper, we give a short account of two specific theoretical frameworks, i.e., the continuum-discretized coupled-channels (CDCC) method for elastic breakup (EBU) calculations and the Ichimura, Austern and Vincent (IAV) model for non-elastic breakup (NEB) [16].

The CDCC approach is an extension of the coupled-channels technique, which incorporates the couplings to unbound resonances and the non-resonant continuum states. This method was first developed aiming to describe the breakup coupling effects on deuteron scattering and later has been applied to the scattering of weakly-bound light heavy ions [5]. In the CDCC treatment, the unbound continuum is discretized into a finite number of states to obtain a tractable problem that may be treated with standard coupled channels techniques. The standard CDCC treats a three-body system, without considering the excitations of the projectile core and the target. It therefore describes the EBU process, where all of the outgoing particles are emitted in their ground states. While CDCC works successfully for weakly bound nuclei that can be described as two-body clusters, it encounters limitations when dealing with unstable weakly bound nuclei where the assumption of an inert core or a two-body cluster structure breaks down. To address this, extensions of CDCC include the effects of core excitation (XCDCC) [17], [18] and the treatment of three-body [19], [20], [21] cluster nuclei.

The CDCC technique is generally effective in describing exclusive breakup reactions. For an inclusive breakup reaction, however, the recorded fragment could be produced through various processes, such as EBU, inelastic breakup (the fragment and/or the target are excited), transfer, and incomplete fusion (ICF, i.e., the other fragment is absorbed by the target, forming a compound nucleus) reactions. Apart from the EBU process, the other contributors are hence referred to as NEB. An accurate calculation of the NEB part is in general not possible [13] owing to the large number of accessible states. Since 1980s, several alternative methods have been proposed and developed. One of these methods, the IAV model, has been revisited recently and successfully applied to several inclusive breakup reactions [22], [23]. The IAV method is based on the participant-spectator model: the recorded fragment is treated as a spectator, while the fragment transferred to or captured by the target is considered the participant particle. The interaction between the participant particle and the target, and therefore the non-elastic cross section, is described using an optical potential.

## 3 Breakup reactions of ${}^{6,7}$Li and ${}^{9}$Be

${}^{6,7}$Li and ${}^{9}$Be are stable but weakly bound nuclei, which are ideal for studies of breakup dynamics at energies close to the Coulomb barrier. They exhibit a two-body and/or three-body clustering structure (α+d for ${}^{6}$Li, α+t for ${}^{7}$Li and α+α+n for ${}^{9}$Be) with binding energies of 1.47, 2.47 and 1.57 MeV, respectively. Moreover, two more decay modes of ${}^{9}$Be were found: ${}^{8}$Be+n and ${}^{5}$He+${}^{4}$He with binding energies of 1.66 MeV and 2.46 MeV, respectively, decaying finally to α+α+n due to the unbound nature of ${}^{8}$Be and ${}^{5}$He [24]. In the following, we will focus on exclusive breakup measurements of these three stable weakly bound nuclei interacted with light, medium-mass and heavy targets, respectively.

### 3.1 Reactions with light targets

**Fig. 2:**
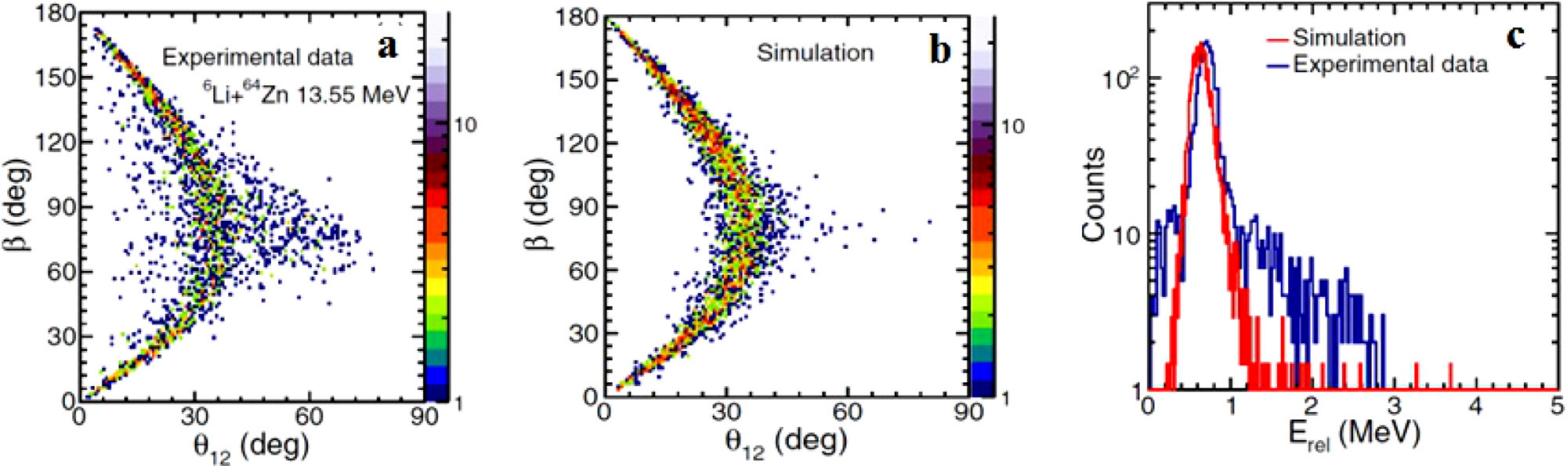
**Comparison of experimental data with simulations for**${}^{6}$Li+${}^{64}$**Zn target at 13.55 MeV.** (a) Experimental β vs $\theta_{12}$ distribution; (b) simulations for ${}^{6}$Li into α and d through the $3^{+}$ resonance; and (c) comparison of simulated (red) and experimental (blue) $E_{\text{rel}}$ distributions. Figures are taken from Ref. [11]. (For interpretation of the references to colour in this figure legend, the reader is referred to the web version of this article.)

Exclusive breakup of ${}^{6,7}$Li on ${}^{58}$Ni and ${}^{64}$Zn have been measured at sub-barrier energies (∼0.95 $V_{\text{B}}$, where $V_{\text{B}}$ denotes the height of the Coulomb barrier) by Kalkal et al. [11]. A detector array with a large angular coverage, BALiN, was used to detect the direct breakup fragments. Several breakup modes were observed, such as α+d and α+p for ${}^{6}$Li and α+α, α+p, α+d and α+t for ${}^{7}$Li. A detailed analysis was performed for the direct breakup reactions, i.e., ${}^{6}$Li into α−d and ${}^{7}$Li into α−t, to distinguish near-target and asymptotic breakup, which is essential to understand the role of breakup in complete fusion (CF) suppression at above-barrier energies. As described above, information of the breakup modes can be obtained from the $E_{\text{rel}}$ distribution and the $\beta \;-\;\theta_{12}$ correlation. In order to further investigate the near-target breakup component, simulations of breakup were carried out using the improved classical three-body dynamical model, PLATYPUS [25]. In the improved version, the lifetimes of the excited states of ${}^{6,7}$Li were specified explicitly, allowing for detailed tracking of the trajectories of each breakup fragment in PLATYPUS. Thus, the positions of breakup could be extracted by comparing the simulations with experimental data. The experimental distributions of β vs $\theta_{12}$ and $E_{\text{rel}}$ of ${}^{6}$Li+${}^{64}$Zn at 13.55 MeV are shown in Fig. 2a, c, respectively, while the PLATYPUS simulations for breakup through the long-lived $3^{+}$ resonant state in ${}^{6}$Li are presented in Fig. 2b, c. One can see that the simulations can reproduce the experimental results reasonably well, indicating that the majority of events (∼93%) in collisions of ${}^{6}$Li with ${}^{58}$Ni and ${}^{64}$Zn are from the asymptotic breakup via the long-lived $3^{+}$ resonance. However, no discernible direct breakup events were observed for ${}^{7}$Li with these light targets, indicating that the dominant component is breakup triggered by transfer reactions.

α−d coincident measurements of ${}^{6}$Li+${}^{59}$Co were performed by Souza et al. [26] at near-barrier energies of 17.4 MeV, 21.5 MeV, 25.5 MeV and 29.6 MeV. The events of breakup via the first $3^{+}$ resonance of ${}^{6}$Li were identified using the $E_{\text{rel}}$ spectra. Angular distributions of the $3^{+}$ resonant breakup were derived and found to be well-reproduced by CDCC calculations. The cross sections of ${}^{6}$Li into α+d were estimated with CDCC calculations. The results suggest that, at energies above the Coulomb barrier, the breakup via the long-lived $3^{+}$ resonant state of ${}^{6}$Li on ${}^{59}$Co contributes less than 50% of the total α−d production. Furthermore, this fraction decreases as the bombarding energy increases.

Exclusive breakup measurement of ${}^{6,7}$Li+${}^{65}$Cu at 25 MeV was performed by Shrivastava et al. [27]. They identified the breakup modes of ${}^{6}$Li through the $3^{+}$ (2.18 MeV) and $2^{+}$ (4.31 MeV) into α+d. The angular distributions of these fragments were reproduced well by CDCC calculations. In the case of ${}^{7}$Li+${}^{65}$Cu, however, besides the observation of α+t breakup mode through the first resonance (4.63 MeV) of ${}^{7}$Li, a large yield of α+d coincident events were detected. Extensive coupled channels Born approximation (CCBA) calculations were performed to further investigate this mechanism. The calculations indicate that the direct transfer of the neutron to the continuum states of ${}^{6}$Li is the dominant procedure. Moreover, the exclusive breakup cross sections of both ${}^{6}$Li and ${}^{7}$Li constitute only a small fraction of the total reaction cross sections. The contributions of exclusive breakup of ${}^{6,7}$Li+${}^{65}$Cu and fusion evaporation are less than 10% and 30%, respectively, to the observed large inclusive α cross section. This suggests that deuteron(triton) capture and/or deuteron(triton) transfer reactions of ${}^{6}$Li(${}^{7}$Li) play a major role.

More complete set of exclusive breakup data of ${}^{7}$Li on a ${}^{93}$Nb target at 24 MeV, 28 MeV and 30 MeV were obtained by Pandit et al. [28]. Coincident events of α+d, α+t and α+α were observed. CDCC and CCBA calculations were performed and compared with the experimental data. The theoretical results confirmed that α+d and α+α are mainly from the direct 1n stripping to the ${}^{6}$Li $3^{+}$ (2.18 MeV) state and the 1p pickup to the ${}^{8}$Be ground state, respectively. The cross sections of the direct and transfer-triggered breakup are comparable. The result also established that the large yield of inclusive α should be resulted from other processes, presumably fusion evaporation and t stripping and/or capture.

### 3.2 Reactions with medium-mass targets

Chattopadhyay et al. performed coincident measurements of ${}^{6}$Li [29] and ${}^{7}$Li [30] interacted with ${}^{112}$Sn. In the ${}^{6}$Li+${}^{112}$Sn experiment, two beam energies of 22 MeV and 30 MeV were used. Besides the breakup through the resonances of $3^{+}$ (2.18 MeV) and $2^{+}$ (4.31 MeV), the breakup mode of ${}^{6}$Li into α+d via its resonance of $1^{+}$ (5.65 MeV) was measured for the first time at 30 MeV. The results suggest that breakup via the $3^{+}$ resonant state of ${}^{6}$Li dominates the total α+d breakup cross section. However, at the lower energy of 22 MeV, the α+d breakup originates only from the direct prompt breakup process. Furthermore, two other major breakup channels were observed at both energies: (i) α+p mode triggered by 1n stripping and (ii) α+α triggered by 1d pickup. The Q value and $E_{\text{rel}}$ spectra demonstrate that α+p breakup arises through the same excited states at both the beam energies. For the α+α channel, however, the breakup at 22 MeV occurs only through the $0^{+}$ state of ${}^{8}$Be, whereas for the case of higher energy 30 MeV, it occurs via both the $0^{+}$ and $2^{+}$ states of ${}^{8}$Be. Compared with α+p and α+α channels, the α+d breakup is the major component at the measured energies.

The measurement of ${}^{7}$Li+${}^{112}$Sn was performed at 30 MeV. In addition to the breakup channel of ${}^{7}$Li into α+t via the first resonance ($7/2^{-}$, 4.63 MeV), the mode of α+t through its second resonance of $5/2^{-}$ (6.68 MeV) was observed for the first time. The probabilities of transfer-triggered breakup via 1n and 2n stripping channels, i.e., (${}^{7}$Li,${}^{6}$Li) and (${}^{7}$Li,${}^{5}$Li) into α+d and α+p, were found to dominate over the direct α+t breakup mode. Moreover, the direct breakup of ${}^{7}$Li→${}^{6}$He+p was observed for the first time with a significant cross section, indicating the importance of the new (${}^{6}$He+p) configuration, which should be considered in understanding the structure and energy levels of ${}^{7}$Li.

**Fig. 3:**
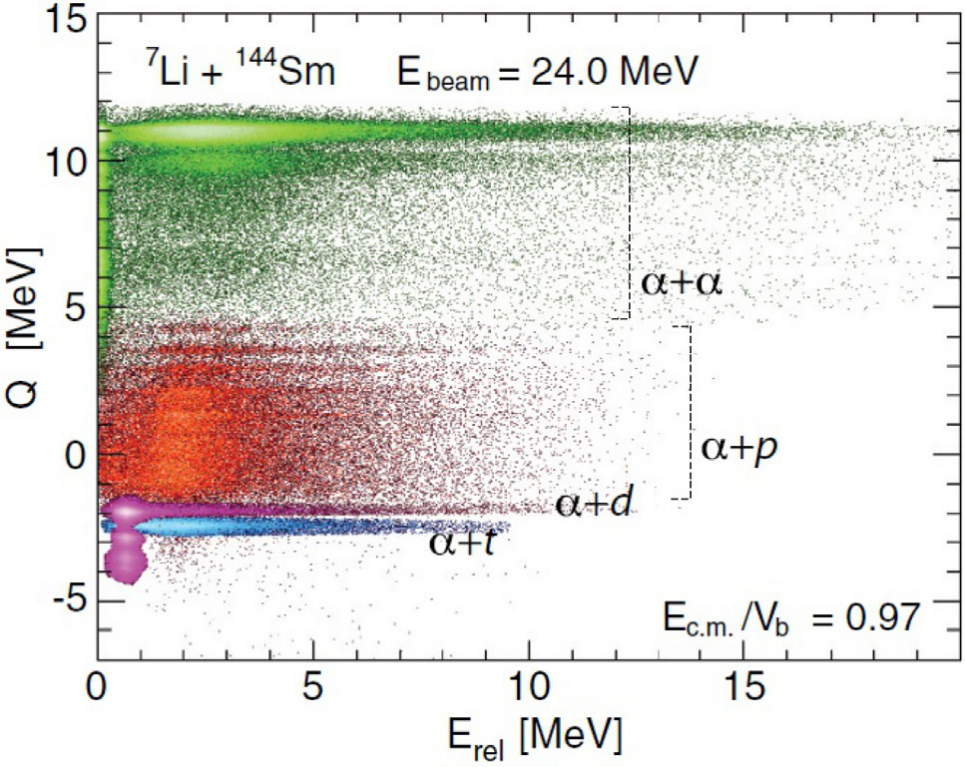
Q vs $E_{\text{rel}}$**two-dimensional spectrum of**${}^{7}$Li+${}^{144}$**Sm at 24 MeV.** The figure is taken from Ref. [31].

Coincident measurements were carried out for ${}^{7}$Li+${}^{144}$Sm at sub-barrier energies (21.5 MeV and 24.0 MeV) by Luong et al. [31]. Different breakup modes, such as α+p, α+d, α+t and α+α, were identified using the Q vs $E_{\text{rel}}$ spectrum (Fig. 3). The Q value reveals the reaction process that triggers the breakup, and $E_{\text{rel}}$ gives the time-scale information of the binary breakup, allowing the separation between prompt and asymptotic breakup. It was found that the breakup of ${}^{7}$Li+${}^{144}$Sm is predominantly triggered by nucleon transfer, with p pickup leading to α+α fragments being the preferred breakup mode.

The exclusive measurements of ${}^{9}$Be on ${}^{144}$Sm and ${}^{168}$Er were performed by Rafiei et al. [8] at near-barrier energies. The energy and angular correlations between the breakup fragments [10] indicate that the breakup mode of ${}^{9}$Be is dominated by the breakup of ${}^{8}$Be into α+α via its ground state, following 1n transfer of ${}^{9}$Be. However, this observed breakup mechanism of ${}^{9}$Be cannot fully explain the suppression of CF at above-barrier energies [32], [33].

### 3.3 Reactions with heavy targets

**Fig. 4:**
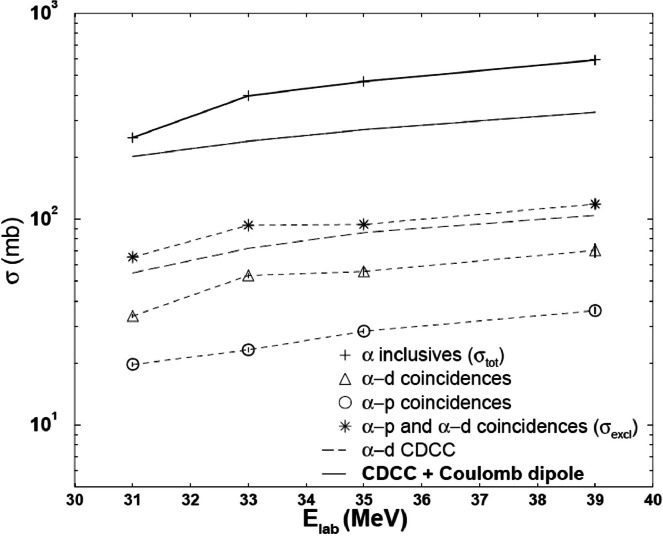
**Inclusive and exclusive total breakup cross sections of**${}^{6}$Li+${}^{208}$**Pb.** The figure is taken from Ref. [34].

Exclusive measurements of ${}^{6,7}$Li+${}^{208}$Pb and ${}^{209}$Bi have been performed by several groups. Signorini et al. [34] measured the breakup of ${}^{6}$Li into α+d and α+p fragments from a ${}^{208}$Pb target at energies ranging from 31 MeV to 39 MeV. The angular distributions of α+d and α+p were derived. The results of ${}^{6}$Li into α+d were reproduced well by CDCC calculations. The excitation functions of exclusive breakup into α+d and α+p, as well as that of the inclusive α are shown in Fig. 4. It can be found that the cross section of breakup into α+d is larger than that of the 1n transfer triggered breakup into α+p. Additionally, the total exclusive cross sections were found to be much smaller than those of the inclusive processes.

Exclusive measurements of the α+d breakup of ${}^{6}$Li+${}^{209}$Bi were performed by Santra et al. [35] at 36 MeV and 40 MeV. The breakup component via the first $3^{+}$ resonant state (2.18 MeV) of ${}^{6}$Li was identified from the $E_{\text{rel}}$ spectra. It was found that this resonant breakup dominates the α+d breakup mode of ${}^{6}$Li+${}^{209}$Bi at the two measured energies. CDCC calculations were performed using the cluster folded potential, and the results reproduce the breakup cross sections of α+d well. Moreover, the α+p breakup followed the 1p stripping of ${}^{6}$Li was observed as well. The cross section of this transfer triggered breakup is comparable to the breakup through the $3^{+}$ resonant state of ${}^{6}$Li, and significantly higher than that of the non-resonant α+d breakup.

**Fig. 5:**
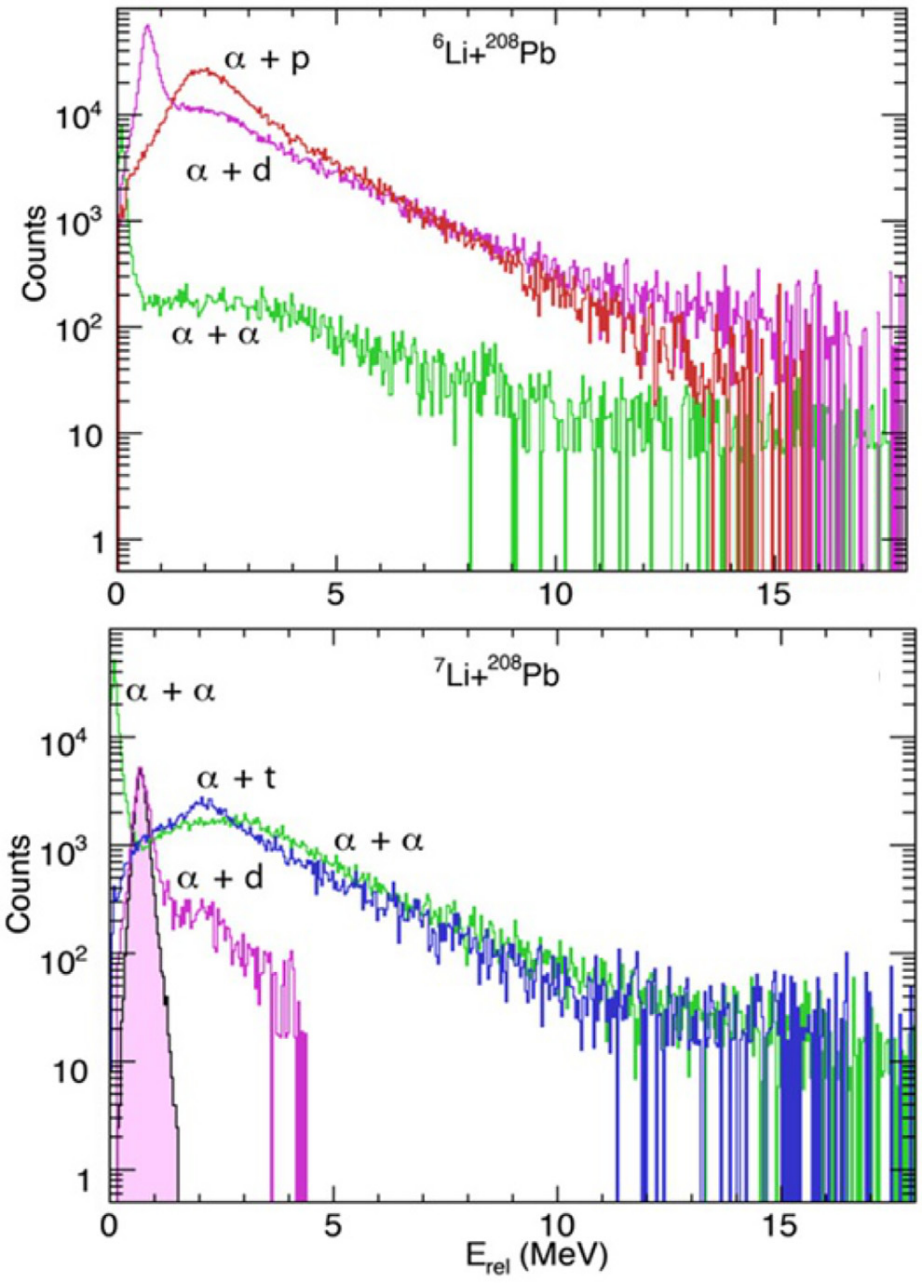
**Relative energy spectra of**${}^{6,7}$Li+${}^{208}$**Pb.** The curves denote the relative energy spectra of breakup fragments of ${}^{6}$Li (upper panel) and of ${}^{7}$Li (lower panel) in collisions with ${}^{208}$Pb at 29.0 MeV. The figure is taken from Ref. [36].

**Fig. 6:**
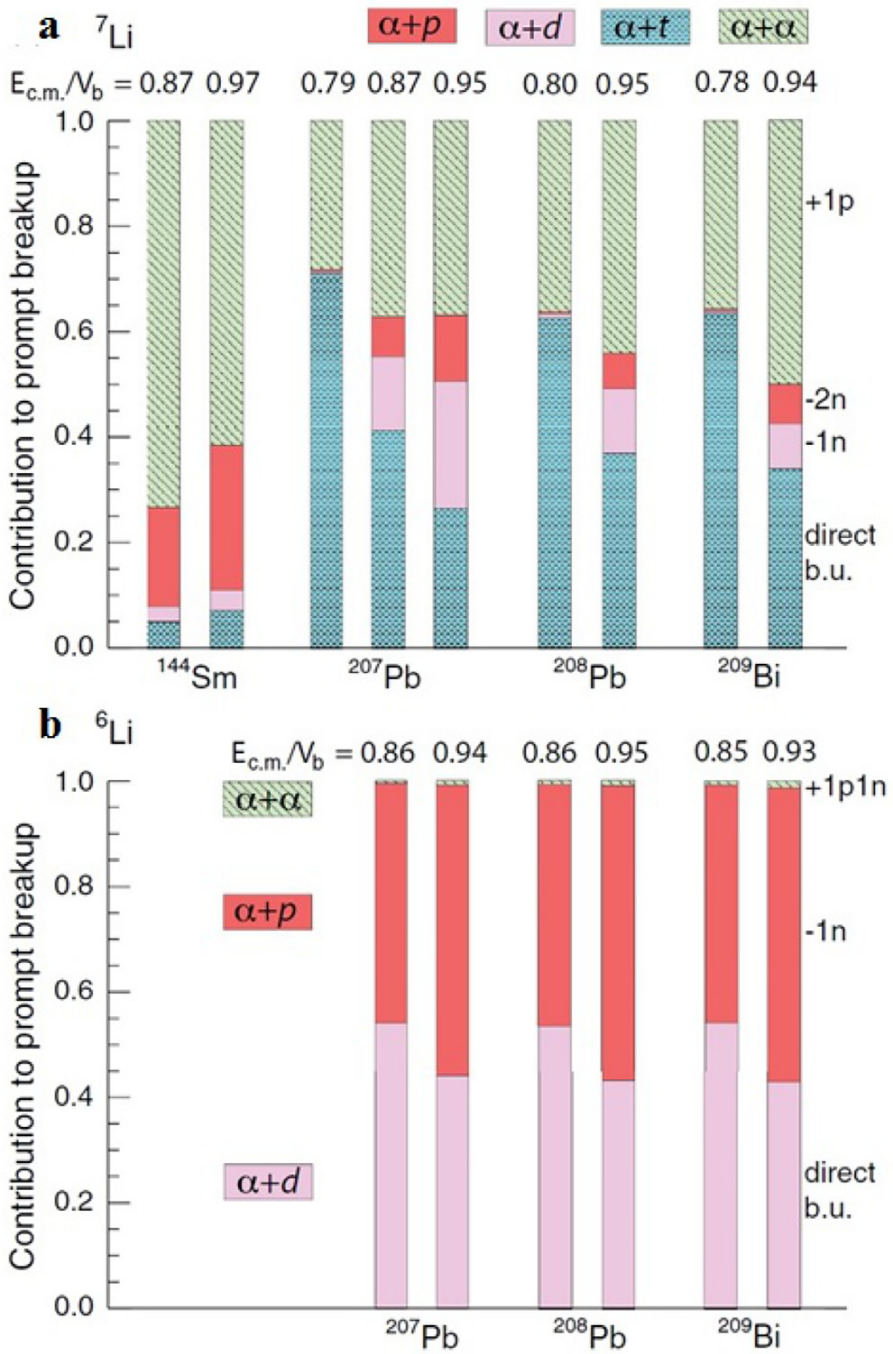
**Relative contributions of various prompt breakup modes.** The histograms show the prompt α+α, α+t, α+d, and α+p breakup to the total prompt breakup of ${}^{6,7}$Li on indicated targets. The figures are taken from Ref. [31].

Luong et al. [36] measured the exclusive breakup of ${}^{6,7}$Li+${}^{208}$Pb at energies below the Coulomb barrier, aiming to investigate the time-scales of breakup of ${}^{6,7}$Li. The reconstructed $E_{\text{rel}}$ spectra of various breakup channels are shown in Fig. 5. For both the ${}^{6}$Li and ${}^{7}$Li reactions, the $E_{\text{rel}}$ spectra for the α+α breakup channel present a sharp peak at 92 keV, corresponding to the long-lived decay of the ${}^{8}$Be ground state. This comprises about half of the total yield of the α+α channel. Regarding the α+d mode, the peak is located at 0.7 MeV, corresponding to the decay via the first excited state of ${}^{6}$Li, which has a relatively long lifetime. These states are populated by the direct excitation of ${}^{6}$Li or through n-transfer in the reactions induced by ${}^{7}$Li. For ${}^{6}$Li, the α+p primarily arises from the 1n-transfer triggered breakup and makes the largest contribution to the prompt breakup of ${}^{6}$Li. In the case of ${}^{7}$Li, breakup into α+t with a wide $E_{\text{rel}}$ distribution is the dominant component, indicating essentially a prompt breakup process. However, the dominant contribution to prompt breakup for ${}^{7}$Li is the prompt breakup of ${}^{8}$Be, i.e., the α+α breakup with higher $E_{\text{rel}}$. Luong et al. further extended the measurements for the ${}^{6,7}$Li+${}^{207,208}$Pb and ${}^{209}$Bi at energies below the Coulomb barrier [31]. They found that breakup is predominantly triggered by nucleon transfer reactions: the α+α breakup mode triggered by p pickup being the preferred channel for ${}^{7}$Li, and the α+p triggered by n stripping for ${}^{6}$Li. Since only the prompt breakup may suppress the CF for energies around and above the barrier, they separated the total prompt breakup components from the delayed breakup based on the $E_{\text{rel}}$ spectra. The relative contributions to prompt breakup by the major identified breakup modes are shown in Fig. 6. It can be seen that, for both ${}^{6}$Li and ${}^{7}$Li, the prompt direct breakup dominates at $E_{\text{c.m.}}/V_{\text{B}}<0.87$. As energies approach the barrier, prompt breakup following nucleon transfer reactions becomes dominant.

**Fig. 7:**
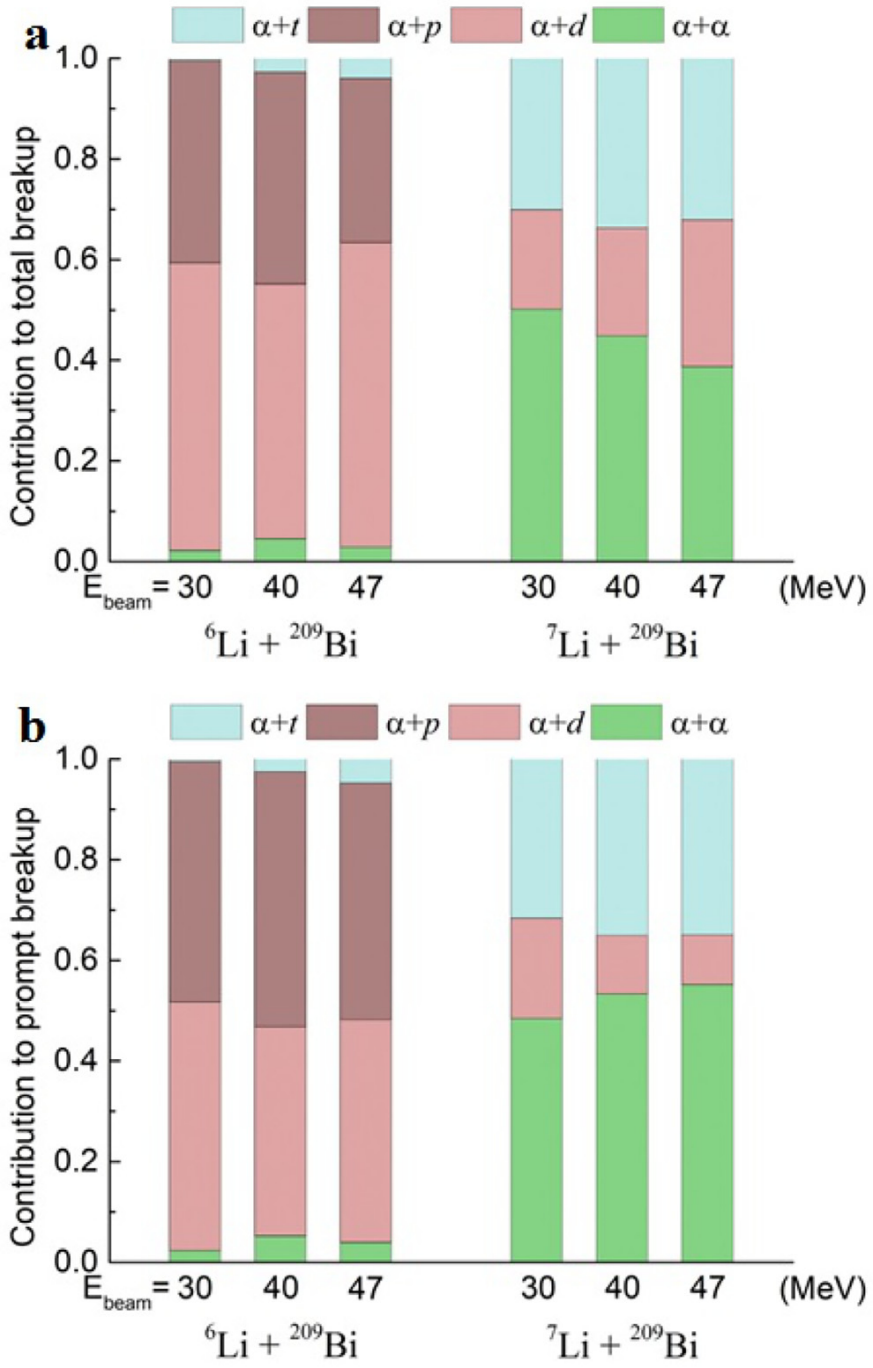
**Relative contributions of various breakup modes of**${}^{6,7}$Li on ${}^{209}$**Bi.** The histograms show the α+α, α+t, α+d, and α+p breakup to (a) the total and (b) the total prompt breakup of ${}^{6,7}$Li on ${}^{209}$Bi at 30, 40 and 47 MeV. The figures are taken from Ref. [37].

**Fig. 8:**
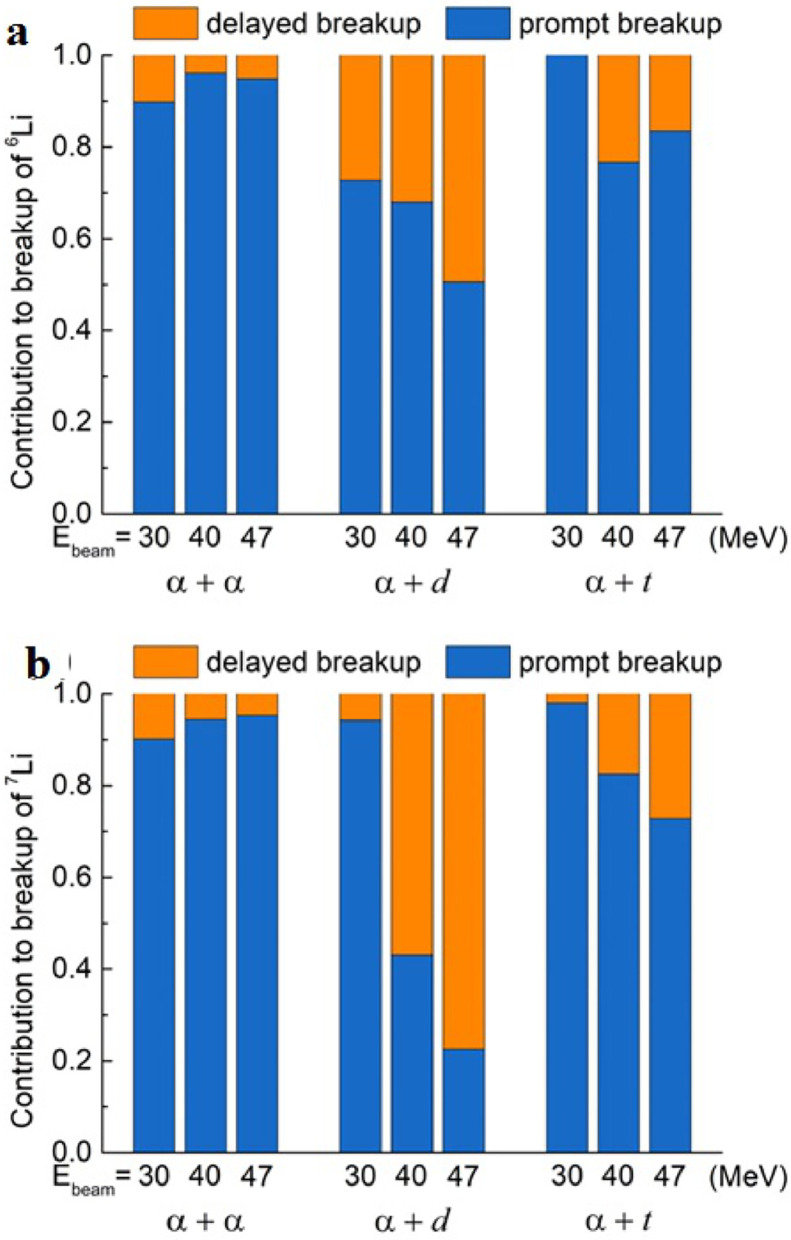
**Relative contributions of various breakup modes of**${}^{6,7}$Li+${}^{209}$**Bi.** The histograms show the prompt and the delayed breakup to the total breakup of ${}^{6,7}$Li+${}^{209}$Bi at indicated energies. The figures are taken from Ref. [37].

The relative probabilities of exclusive breakup channels in reactions of ${}^{6,7}$Li with ${}^{209}$Bi were investigated in detail by Yao et al. [37] at energies (30, 40 and 47 MeV) around and above the Coulomb barrier. Various breakup modes, like α+p, α+d, α+t and α+α were distinguished based on Q values and $E_{\text{rel}}$ distributions of the breakup fragments. Comparing with the results of Luong et al. [31] at sub-barrier energies, the α+t breakup mode of ${}^{6}$Li following 1n pickup was observed at energies above the Coulomb barrier. The prompt and delayed breakup components were further separated from the $E_{\text{rel}}$ spectra. The relative contributions of the observed breakup modes to the total breakup are shown in Fig. 7 a. For reactions induced by ${}^{6}$Li, the contribution of direct breakup into α+d is more significant than the α+p breakup following 1n-stripping. Moreover, the α+t and α+α modes only contribute small parts of the total yields. For the case of ${}^{7}$Li, the major contribution to the total breakup cross section is the α+α breakup of ${}^{8}$Be, triggered by 1p pickup reaction. The contributions of ${}^{7}$Li direct breakup into α+t and the 1n stripping triggered breakup into α+d are comparable. The relative contributions to the prompt breakup, which is essential to understand the influence of breakup on the CF suppression, are presented in Fig. 7b. In reactions of ${}^{6}$Li, the α+p breakup channel, rather than the α+d process, becomes the major contributor of the total prompt breakup cross section. For reactions induced by ${}^{7}$Li, the prompt α+α breakup provides the major contribution to prompt breakup, and it becomes more important as the bombarding energy increases. Shown in Fig. 8 are the relative probabilities of prompt and delayed breakup for different modes in reactions of ${}^{6,7}$Li with ${}^{209}$Bi. The delayed breakup played a minor role in α+α and α+t breakup modes for ${}^{6}$Li and ${}^{7}$Li. However, for the α+d channel, the delayed breakup becomes more important as the incident energy increases due to the existence of the long-live $3^{+}$ resonant state in ${}^{6}$Li.

In Ref. [8], besides ${}^{9}$Be+${}^{144}$Sm and ${}^{168}$Er discussed in the previous section, coincident measurements of ${}^{9}$Be+${}^{196}$Pt, ${}^{208}$Pb and ${}^{209}$Bi were performed as well at energies below the barrier. The $E_{\text{rel}}$ distribution indicates that ${}^{9}$Be mainly breaks up into α+α through the 1n stripping reaction. The experimental data were reanalyzed by Cook et al. [10] to investigate the lifetime effects in breakup. It was found that the angular and energy correlations between the breakup fragments present the sensitivity even to the short-lived $2^{+}$ state of ${}^{8}$Be triggered by 1n transfer from ${}^{9}$Be.

### 3.4 Summary of breakup mechanisms of ${}^{6,7}$Li and ${}^{9}$Be

**Fig. 9:**
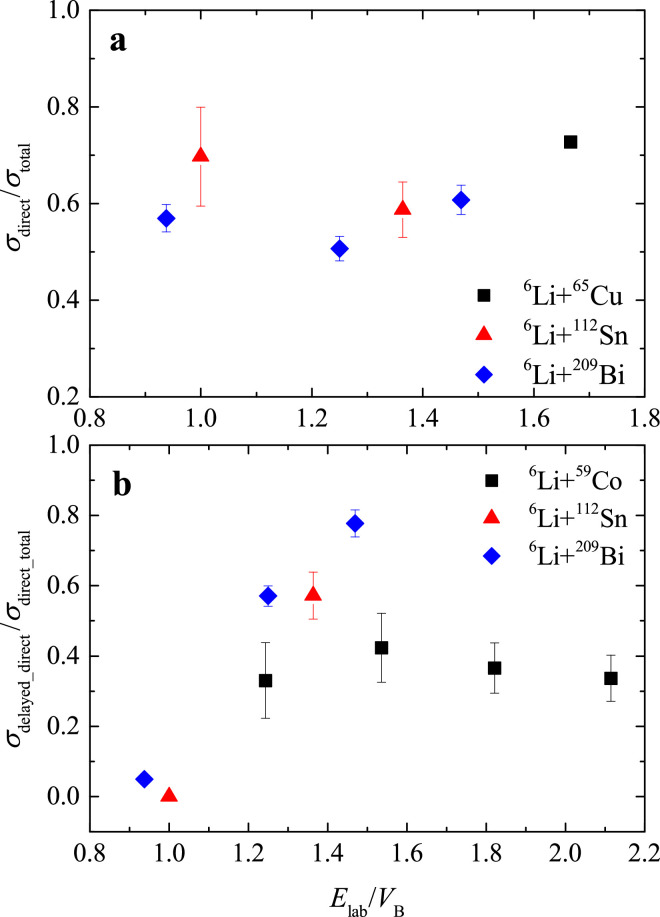
**Relative contributions of various breakup modes of**${}^{6}$**Li.** The histograms show the direct breakup to the total breakup of ${}^{6}$Li (a) and the delayed breakup of ${}^{6}$Li via the $3^{+}$ resonant state to the total direct breakup (b) at indicated energies for different targets.

For reactions induced by ${}^{6}$Li with various targets, several breakup modes, i.e., direct breakup into α+d and transfer-reaction triggered breakup into α+p and α+α, have been observed. The α+t channel following the 1n pickup reaction of ${}^{6}$Li, however, was found only for the ${}^{6}$Li+${}^{209}$Bi [37] system at energies above the barrier. The contributions of direct breakup of ${}^{6}$Li to the total breakup cross section for some typical targets are shown in Fig. 9a. One can see that this contribution ranging from 50% to 80%, remains relatively consistent regardless of the target mass and the incident energy. Furthermore, the ratio of the yield of ${}^{6}$Li delayed breakup via the long-lived $3^{+}$ resonant state to the total direct breakup is presented in Fig. 9b. For the light target ${}^{59}$Co, the contribution of ${}^{6}$Li $3^{+}$ resonant breakup stabilizes at about 36% at energies above the Coulomb barrier. For heavier targets of ${}^{112}$Sn and ${}^{209}$Bi, however, a strong energy dependence is observed: a very small fraction of the $3^{+}$ resonant breakup is obtained at energies around the below the barrier, indicating the predominance of prompt direct breakup of ${}^{6}$Li. As the bombarding energy increases in the above barrier region, the delayed breakup becomes more important and eventually becomes the dominant component when the energy exceeds 1.3 times $V_{\text{B}}$. It might due to the relatively strong Coulomb interaction from the heavier target, which enhances the probability of ${}^{6}$Li excited to its first resonance state.

No direct breakup of ${}^{7}$Li into α+t was observed for ${}^{7}$Li interacts with light targets of ${}^{58}$Ni and ${}^{64}$Zn at sub-barrier energies [11]. However, in the case of ${}^{7}$Li+${}^{65}$Cu [27], the α+t breakup mode was found at 25 MeV, above the Coulomb barrier ($∼1.6V_{\text{B}}$). As the target mass increases, such as in ${}^{7}$Li+${}^{144}$Sm [31], ${}^{208}$Pb [31] and ${}^{209}$Bi [37], direct breakup of ${}^{7}$Li was observed at energies below and above the barrier. The contributions of direct breakup to the total breakup of ${}^{7}$Li+${}^{65}$Cu and ${}^{209}$Bi are around 31% at energies near and above the Coulomb barrier, which is significantly smaller than that of ${}^{6}$Li reaction systems. This is mainly because that, the breakup threshold of ${}^{6}$Li (1.47 MeV) is lower than that of ${}^{7}$Li (2.47 MeV). Therefore, the breakup mechanism of ${}^{7}$Li is predominantly driven by transfer-triggered breakup rather than direct breakup. The probabilities of various transfer-triggered breakup strongly depend on the transfer Q value and the structure of the target.

Compared to ${}^{6}$Li and ${}^{7}$Li, the coincident data for ${}^{9}$Be are relatively scarce. The available data indicate that the α+α breakup mode, following the 1n stripping of ${}^{9}$Be, dominates the total breakup yield.

For ${}^{6,7}$Li and ${}^{9}$Be systems, the observed exclusive α yield is much smaller that that of the inclusive α. Therefore, the major contribution of α may be from processes like breakup fragment captured by the target or cluster transfer.

## 4 Breakup reactions of unstable nuclei

When nuclei move away from the valley of stability in the nuclide landscape, they become unstable. This instability is characterized by extremely low binding energies and hence the extended nuclear matter distributions. Exotic nuclear structure, like, halo, has been discovered in nuclei close to the drip line. The term “halo” was suggested by Hansen and Jonson [38] to describe the long tail observed in the density distribution of a nucleus. The first experimental evidence of the existence of halo was reported by Tanihata et al. in 1985 [6] in the neutron drip line nucleus ${}^{11}$Li. After that, several more neutron halo nuclei have been confirmed experimentally in the vicinity of the neutron drip line, such as ${}^{6}$He, ${}^{11}$Be and ${}^{22}$C [2]. However, owing to the confining of the Coulomb barrier, proton halo nuclei are less common [2]. It is well known that, reaction dynamics is closely related to nuclear structure. Therefore, the reaction mechanisms induced by these unstable exotic nuclei have become one of the most popular topics in nuclear physics.

Kolata et al. [39] have published a valuable review article that discusses reactions induced by radioactive beams at energies close to the Coulomb barrier. The article covers research on elastic scattering, direct and fusion reactions published from 2006 to 2015. In this section, we will focus on recent breakup data of unstable nuclear systems that were not included in Ref. [39].

### 4.1 Breakup reactions of neutron-rich nuclei

#### 4.1.1 Breakup of ${}^{8}$Li

Although the short-lived radioactive nucleus ${}^{8}$Li is not considered to be exotic, it has low neutron separation energy threshold of 2.03 MeV, indicating a weakly bound nature. Cook et al. [40] performed the coincident measurements of charged particles for ${}^{8}$Li+${}^{209}$Bi at above-barrier energies (1.27-1.36$V_{\text{B}}$) to study the breakup modes of ${}^{8}$Li. The Q-value spectrum was used to distinguish the production of ${}^{7}$Li between ${}^{8}$Li direct breakup and neutron stripping. By integrating the angular distributions, the cross sections of ${}^{7}$Li formation via neutron stripping and direct breakup were determined as 16.1±1.7 mb and 219±5 mb, respectively. Coincidence α+α, α+t, α+d and α+p pairs were observed as well. Remarkably, ${}^{8}$Li shows as much diversity in breakup modes as was observed with ${}^{6,7}$Li. It was found that, the cross sections of α+d, α+α, and α+p are σ=6.2±0.9 mb, 5.6±0.7 mb, and 0.9±0.4 mb, respectively, with α+t production being dominant (σ= 19±2 mb). A detailed analysis was performed for the α+t mode, as it is the dominant breakup channel which may suppress the CF cross section. The measured α+t $E_{\text{rel}}$ spectrum shows a well-defined peak at 2.19 MeV, corresponding to the breakup of ${}^{7}$Li from its relatively long-lived $7/2^{-}$ resonant state ($E_{\text{x}}$=4.652 MeV). A classical dynamical simulation for the α+t breakup was performed to estimate the contribution of prompt breakup. The result indicates that 7±1% of α+t breakup mode occurs prior to the distance of closest approach, which hence cannot suppress the CF cross section of ${}^{8}$Li significantly.

#### 4.1.2 Breakup of ${}^{11}$Be

The ${}^{11}$Be nucleus has a $J^{\pi }\;=\;1/2^{+}$ ground state with a neutron binding energy of 0.501 MeV, presenting a one-neutron halo structure. The ground state is known to contain admixtures of s and d neutron configurations, with the latter associated with excited components of the ${}^{10}$Be core. Notably, ${}^{11}$Be is the only halo nucleus currently known to have a strongly deformed core [41]. The first excited state of ${}^{11}$Be ($E_{x}$=320 keV, $J^{\pi }\;=\;1/2^{-}$) is a bound state. The reduced dipole transition probability between the ground state and the first excited state is B(E1)=0.116(12) $e^{2}fm^{2}$ [42], which is one of the largest known values. Consequently, these unusual features of ${}^{11}$Be strongly affect the reaction dynamics at bombarding energies close to the Coulomb barrier [39].

An inclusive breakup measurement was performed for the ${}^{11}$Be+${}^{64}$Zn system at $E_{c.m.}=$24.5 MeV [43]. The data from this experiment have been included and discussed in Ref. [39]. Recently, these inclusive breakup observables of this experiment were reexamined to investigate the contributions of EBU and NEB, as well as the post-acceleration in the energy distribution of ${}^{10}$Be [44]. CDCC and XCDCC calculations, with the latter considering effects arising from ${}^{10}$Be deformation, predict similar EBU cross sections, which contribute ∼80% to the total yield of ${}^{10}$Be. According to the IAV model calculation, the remaining contribution arises from the NEB process. The position of the centroid of the ${}^{10}$Be energy distribution as a function of the laboratory angle could be well reproduced by kinematical calculations including the post-acceleration effect. These results indicate a complicated mechanism of breakup reaction.

Pesudo et al. [45] performed the first measurement of ${}^{11}$Be+${}^{197}$Au scattering at $E_{\text{lab}}=31.9\;\text{MeV}$ and 39.6 MeV, which are, respectively, below and around the Coulomb barrier ($V_{\text{B}}=40$ MeV). Besides the silicon detector array used to identify the charged particle of ${}^{10}$Be and ${}^{11}$Be, the gamma detector array TIGRESS [46] was installed to record the 320 keV gamma ray from the de-excitation of the first excited state. For the breakup measurement, due to the lack of neutron detection, only the inclusive angular distributions of ${}^{10}$Be were derived at the two measured energies. CDCC and XCDCC calculations were performed and compared with the experimental results. In the XCDCC calculation, the structure of ${}^{11}$Be is described using a particle-plus-core model, with the consideration of the ground state and the first excited state in ${}^{10}$Be. The results show that, only the XCDCC calculations could reproduce the elastic, inelastic, and breakup differential cross sections, indicating that the dynamical core polarization is essential to understand the reaction mechanisms induced by ${}^{11}$Be.

Inclusive breakup measurements of ${}^{11}$Be on a ${}^{208}$Pb target at energies of 140 [47] and 210 [48] MeV were performed by Duan et al. at HIRFL-RIBLL (Heavy-Ion Research Facility in Lanzhou and Radioactive Ion Beam Line in Lanzhou, China). Despite these energies being 3.5 and 5.2 times higher than the Coulomb barrier, we discuss these data as well since they provide interesting complementary information. The angular distributions of quasielastic scattering of these two energies present significant suppression of the Coulomb-nuclear interference peak. Theoretically, both CDCC and XCDCC calculations are able to successfully reproduce the quasielastic scattering data. However, calculations omitting the couplings to the continuum states cannot describe the structure of the angular distributions well. This further confirms that the strong breakup coupling effect, which has been mostly found for halo nuclei at energies close to the Coulomb barrier, persist in the reactions induced by ${}^{11}$Be at incident energies several times higher than that of the Coulomb barrier. Regarding the inclusive angular distributions of ${}^{10}$Be, CDCC and XCDCC results are nearly identical with each other, suggesting that core excitations are not significant for breakup cross section in the direct reaction. Moreover, CDCC (XCDCC) calculations could reproduce the main peak located in the forward angular region in the inclusive angular distributions of ${}^{10}$Be, while fail to describe the bump at $\theta_{\text{lab}}>10^{\circ }$. IAV calculations were performed to estimate the contribution of NEB. It was found that the sum of EBU from CDCC and NEB from IAV calculation agrees rather well with the inclusive data. This result indicates that the contribution of NEB cannot be neglected for ${}^{11}$Be+${}^{208}$Pb. Furthermore, the energy distributions of the ${}^{10}$Be core at incident energy of 140 MeV were investigated. It was found that only the kinematical model which considers the strong Coulomb interaction between the breakup fragment and the heavy target, i.e., the post-acceleration effect, could reproduce the position of the centroid of the ${}^{10}$Be energy distributions. This result suggests that the near-target prompt breakup is the dominant breakup mode of ${}^{11}$Be+${}^{208}$Pb.

### 4.2 Breakup reactions of proton-rich nuclei

#### 4.2.1 Breakup of ${}^{8}$B

The proton drip line nucleus ${}^{8}$B has extremely low proton separation energy of merely 138 keV. More intriguing, it is one of the few observed cases whose ground state exhibits a proton halo [49], [50], [51]. Therefore, ${}^{8}$B offers an excellent opportunity to investigate the breakup dynamics induced by proton halo nuclei.

Several inclusive breakup data sets of ${}^{8}$B have been reported recently. Spartà et al. [52] performed the measurement of ${}^{8}$B+${}^{64}$Zn at an energy around 1.5 times the Coulomb barrier at HIE-ISOLDE CERN. The inclusive angular ${}^{7}$Be distribution was compared with CDCC calculations, and the result indicated that EBU dominates at small angles, whereas NEB becomes non-negligible at larger angles. The energy distribution of ${}^{7}$Be was extracted, trying to gain information into breakup dynamics and postacceleration effects. However, the calculations suggest that the dynamics of ${}^{8}$B breakup is rather complicated: CDCC and DWBA (Distorted-Wave Born Approximation) calculations show that various effects could affect the ${}^{7}$Be energy distribution, such as the different Coulomb multipoles in the breakup process and their interference, as well as nuclear effects. To better understand all these effects, it is necessary to perform exclusive measurements of all outgoing fragments of ${}^{8}$B.

The inclusive measurement of ${}^{8}$B+${}^{208}$Pb was performed by Pakou et al. at a deep sub-barrier energy of 30 MeV [53] at the TwinSol facility of the University of Notre Dame. The inclusive angular distribution of the breakup fragment ${}^{7}$Be could be reproduced successfully by CDCC calculations. By integrating the data and extrapolating to more forward and backward angles based on the CDCC calculation, the experimental breakup cross section was determined as 326±84 mb, which exhausts all of the total reaction cross section for the system ${}^{8}$B+${}^{208}$Pb at the energy well below the barrier. It is worth noting that this breakup cross section may include incomplete fusion (proton capture) which cannot be distinguished from the inclusive measurement.

The inclusive angular distribution of ${}^{8}$B on a ${}^{208}$Pb target at a high energy of 238 MeV (4 times the Coulomb barrier) was reported by Wang et al. [54] at HIRFL-RIBLL. The CDCC calculation reproduces well the angular distribution of ${}^{7}$Be at very forward angular region up to 7${}^{\circ }$, but it underestimates the bump structure at larger angles. The contribution of the NEB process was estimated by the IAV model calculation. The CDCC+IAV result describes rather well the structure of the data. The theoretical analysis also indicates that, the EBU mechanism dominates the inclusive ${}^{7}$Be yield, with a small but non-negligible contribution from NEB processes.

**Fig. 10:**
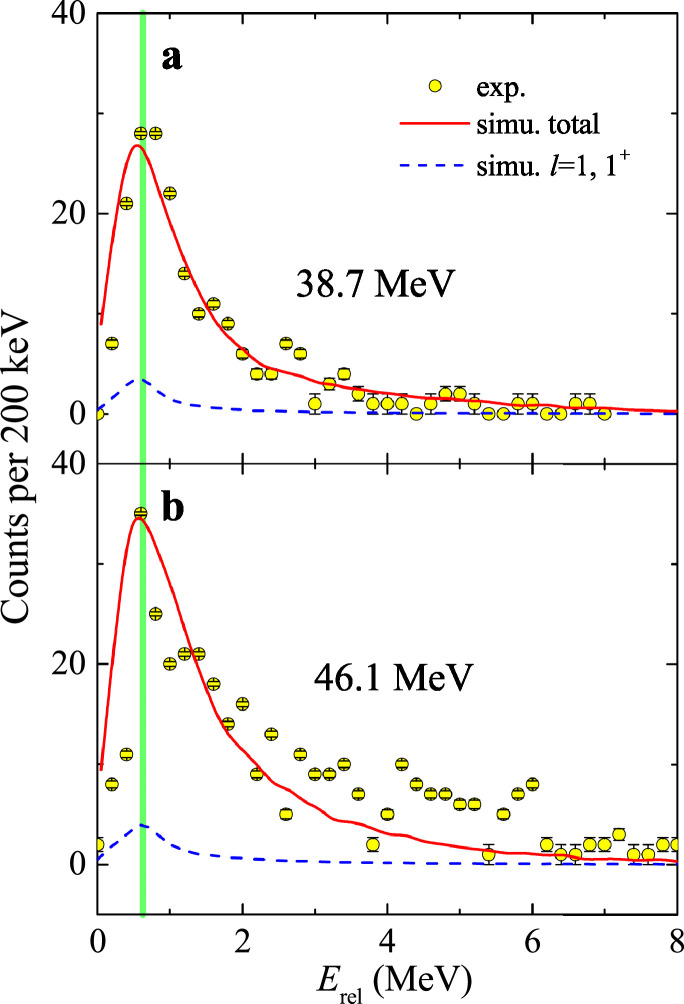
$E_{\text{rel}}$**distributions for**${}^{8}$B+${}^{120}$**Sn.** The measured $E_{\text{rel}}$ distributions for breakup fragments ${}^{7}$Be and proton at (a) 38.7 MeV and (b) 46.1 MeV, and compared with the simulation results (solid curves). The dashed curves denote simulation results of p−wave $1^{+}$ state. The vertical line represents the expected location of the first $1^{+}$ resonant state of ${}^{8}$B. The figures are taken and modified from Ref. [55].

**Fig. 11:**
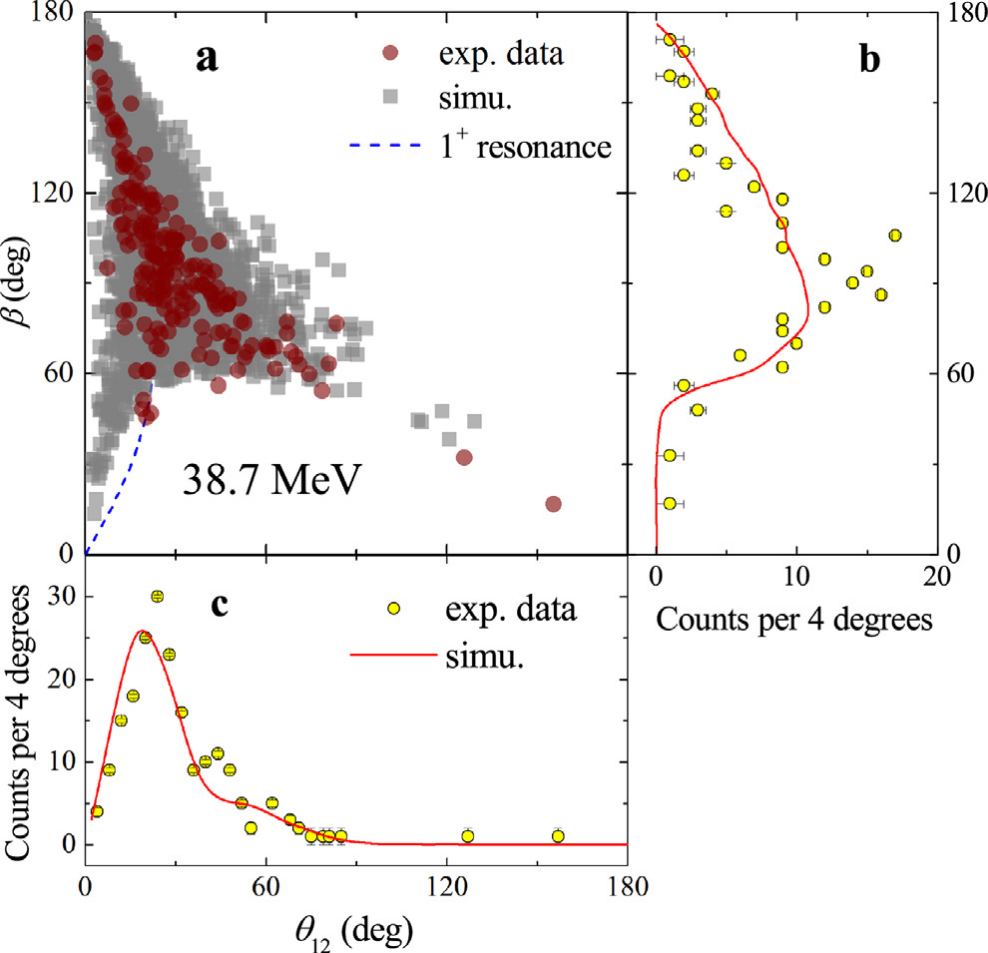
**Angular correlations of the breakup fragments of**${}^{8}$B+${}^{120}$**Sn.** Comparison of experimental data (circles) with simulations (squares) for β vs $\theta_{12}$ (a) at 38.7 MeV. The dashed curve shows the expected $\beta \;-\;\theta_{12}$ correlation assuming asymptotic breakup from the $1^{+}$ resonance of ${}^{8}$B. The projections of β and $\theta_{12}$ are shown in panels (b)and (c), respectively. The solid curves denote the simulations results. The figures are taken and modified from Ref. [55].

The first exclusive breakup measurement of ${}^{8}$B on a medium-mass target ${}^{120}$Sn was performed by Yang et al. [55] at two energies (37.8±0.5 MeV and 46.1±0.6 MeV) around the Coulomb barrier. The experiment was performed at the low-energy radioactive-ion beam facility CNS Radioactive Ion Beam separator (CRIB) [56] of the Center for Nuclear Study (CNS), the University of Tokyo. With the help of high-efficient silicon detector array STARE [37], the correlations between the breakup fragments of ${}^{7}$Be and proton were successfully derived. To find the efficiency of coincident measurement realistically, a novel simulation approach based on the detailed outputs of CDCC was established. First, five-fold differential cross sections, $d\sigma /d\Omega_{{}^{7}\text{Be}}dE_{{}^{7}\text{Be}}d\Omega_{p}$, were produced from the CDCC calculations [57]. The Markov chain Monte Carlo (MCMC) method [58] was then used to sample from the five-fold parameter space. Assuming $\phi_{{}^{7}\text{Be}}$ with a flat distribution, the kinematic parameters of $E_{{}^{7}\text{Be}}$, $\theta_{{}^{7}\text{Be}},\theta_{p}$ and $\phi_{p}$ in the laboratory frame could be obtained from the MCMC sampling. The only unknown parameter, the energy of proton $E_{p}$, can be hence determined based on the energy and momentum conservations of the breakup reaction. With the complete kinematics information of each fragment and the geometry of detector array, the coincident detection efficiency was deduced finally from a Monte Carlo simulation. The angular distributions of inclusive and exclusive breakup are found to be almost identical at the two measured energies, offering clear experimental evidence that EBU dominates the ${}^{7}$Be production mechanism. CDCC and IAV model calculations were performed to estimate the contributions from EBU and NEB. It was found that, the exclusive and inclusive breakup angular distributions could be reproduced well by CDCC and CDCC+IAV calculations, respectively. The cross sections of EBU ($\sigma_{\text{EBU}}$) and NEB ($\sigma_{\text{NEB}}$) are, respectively, 351.5 (420.5) mb and 78.3 (91.4) mb for 38.7 (46.1) MeV, indicating clearly that the NEB contribution is just minor (∼18% of the total ${}^{7}$Be yield). The reconstructed $E_{\text{rel}}$ distributions of ${}^{7}$Be+p at 38.7 and 46.1 MeV are shown by the circles in Fig. 10 a and b, respectively, where the solid curves denote the simulation results. Overall, the simulations successfully reproduce the structure of the experimental data at both energies. Moreover, a distinctive peak at around 0.6 MeV is observed in Fig. 10, which is very close to the location of the first resonance of ${}^{8}$B ($E_{x}$=0.77 MeV, $J^{\pi }$=1${}^{+}$, Γ=35.6 keV), as indicated by the vertical line. CDCC calculations were performed to highlight the relative contribution of the first resonance. However, the results indicate that, the contributions of this resonant state are only (4.4±2.0)% and (3.8±2.5)% at 38.7 and 46.1 MeV. The small fraction of the 1${}^{+}$ resonant state contribution reveals a prompt breakup mechanism of ${}^{8}$B. The measured $\theta_{12}\;-\;\beta$ correlation at 38.7 MeV is shown in Fig. 11a. and the projections of β and $\theta_{12}$ are shown in Fig. 11b and c, where the squares and solid curves denote the simulation based on the CDCC calculations. One can see that the simulations reasonably reproduced the experimental data. The expected correlation between β and $\theta_{12}$ [9] assuming the asymptotic breakup through the $1^{+}$ resonance of ${}^{8}$B is shown by the dashed curve in Fig. 11a. It can be seen that a majority of the events deviate from the curve, further confirming the predominance of the prompt breakup. Furthermore, the strongly forward-peaked $\theta_{12}$ distribution presented in Fig. 11c suggests that most of the breakup occurs in the outgoing trajectory of ${}^{8}$B, in which case, the initial velocities of the fragments are in the same direction as the Coulomb interaction from the target nucleus, leading to a small $\theta_{12}$.

#### 4.2.2 Breakup of ${}^{17}$F

The proton drip line nucleus ${}^{17}$F has a breakup threshold of 0.6 MeV into ${}^{16}$O+p. The first excited state ($E_{\text{x}}=495$ keV, $J^{\pi }\;=\;1/2^{+}$) of ${}^{17}$F has been reported to present a proton halo structure, which is bound by only 105 keV.

**Fig. 12:**
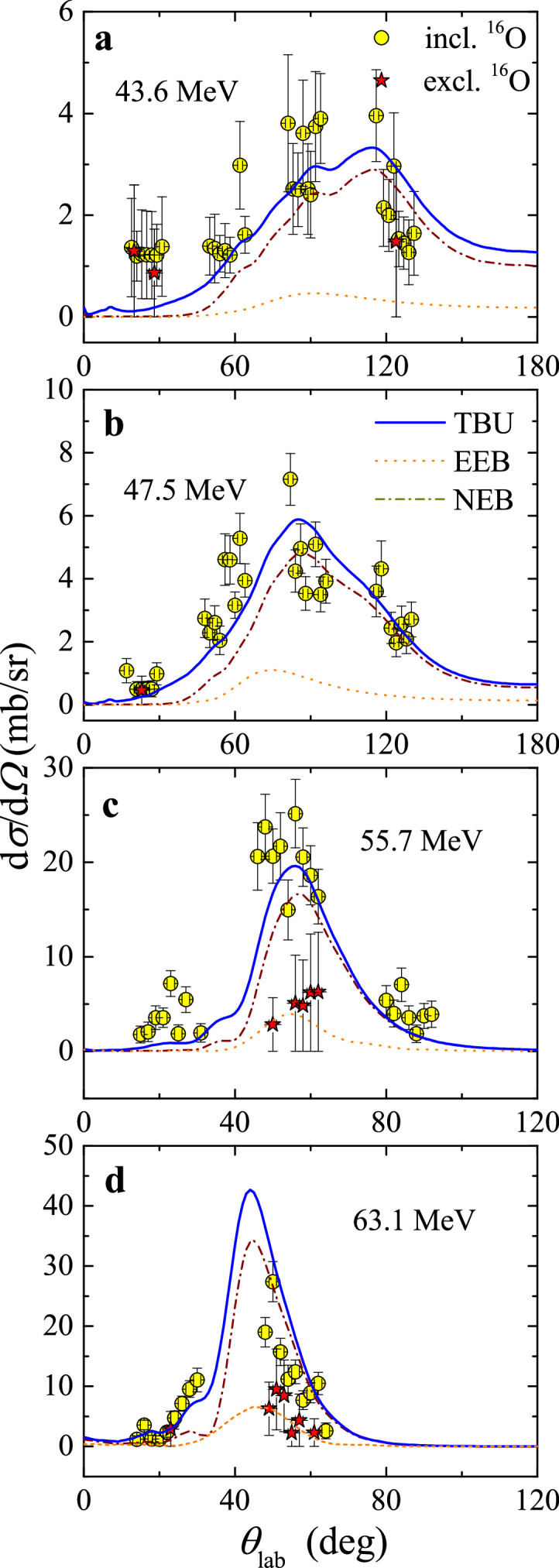
**Angular distributions of**${}^{17}$F+${}^{58}$**Ni.** Angular distributions of exclusive (stars) and inclusive (circles) breakup of ${}^{17}$F+${}^{58}$Ni at four measured energies. The dotted, dash-dotted, and solid lines denote the results of EBU (CDCC), NEB (IAV model), and their sum (TBU), respectively. The figures are taken and modified from Ref. [59].

Recently, Yang et al. [59] performed the complete kinematics measurement of ${}^{17}$F+${}^{58}$Ni at four energies, 43.6±0.7 MeV, 47.5±0.7 MeV, 55.7±0.8 MeV and 63.1±0.9 MeV, near the Coulomb barrier at CRIB. To identify the relatively heavy breakup fragment ${}^{16}$O with low energies, a Multi-layer Ionization-chamber Telescope Array (MITA) [60] was developed. Based on the powerful capability of particle identification and large angular coverage of MITA, information of the nearly full reaction channels, i.e., scattering, exclusive and inclusive breakup, and the fusion reactions, were derived simultaneously for the first time. The angular distributions of exclusive and inclusive breakup of ${}^{17}$F+${}^{58}$Ni at the four measured energies are shown in Fig. 12. CDCC and IAV model calculations were performed to interpret the data, and the corresponding results are shown in Fig. 12 as well. One can find that CDCC calculations reasonably reproduce the exclusive ${}^{16}$O data, and the total breakup (TBU), which is defined as the sum of EBU and NEB, describes properly the structure of the inclusive data. Notably, the NEB component significantly contribute to the inclusive breakup cross section.

### 4.3 Summary of breakup mechanisms of unstable nuclei

**Fig. 13:**
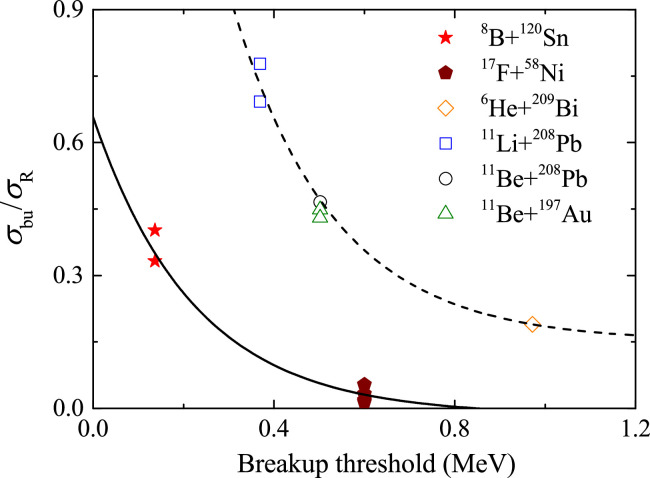
**Ratio of breakup cross section to the total reaction cross section vs. the breakup threshold of indicated systems.** The empty and solid symbols denote the results of neutron- and proton-halo systems, respectively. The lines are used to guide the eyes.

Reactions induced by proton- and neutron-rich nuclei at near-barrier energies exhibit rather different dynamics due to the Coulomb polarization effect: owing to the Coulomb field of the target, the valence proton of the proton-halo nucleus is more likely to be on the side of the projectile core facing away from the target [61], [62]. In semiclassical terms, such Coulomb polarization favors neutrons in the halo residing between the core and the target, which then enhances the transfer probabilities. This effect has been confirmed in reactions induced by ${}^{6}$He: the coincident measurement of alphas and neutrons for the neutron halo system ${}^{6}$He+${}^{209}$Bi [63] established that the 2n-transfer, rather than breakup, was the predominant direct process. However, for the cases of ${}^{11}$Li and ${}^{11}$Be, which possess much lower breakup thresholds, CDCC calculations suggest that the elastic breakup dominates the inclusive breakup cross sections. For proton-rich nuclei, distinct reaction dynamics were found for ${}^{8}$B and ${}^{17}$F reaction systems: for the proton halo nucleus ${}^{8}$B, the exclusive breakup data clearly indicate that the EBU dominates the direct reaction mechanism, while non-elastic breakup becomes the major component for ${}^{17}$F. To gain a further insight into this phenomenon, the ratio of breakup cross section to total reaction cross section vs. the breakup threshold of the indicated systems are presented in Fig. 13, where the results of proton- and neutron-halo systems are respectively presented by solid and empty symbols. For the breakup cross section, the exclusive breakup results are adopted when they are available, otherwise, the theoretical results from CDCC calculations are used for the discussion. As shown in Fig. 13, the breakup probability increases as the breakup threshold decreases. Interestingly, proton halo systems show a smaller breakup probability than the neutron halo systems for the same breakup threshold. That is mainly owing to the additional Coulomb barrier, which does not exist for neutron. Therefore, the effective breakup threshold for proton halo nuclear systems should account for the proton separation energy and the height of the Coulomb barrier between the valence proton and the core [64], [65]. Further experimental and theoretical investigation is deserved to understand the difference between the proton- and neutron-halo reaction systems systematically and comprehensively.

## 5 Conclusions and outlook

We have presented an account of recent experimental works on breakup reactions induced by stable weakly bound nuclei ${}^{6,7}$Li and ${}^{9}$Be, as well as unstable exotic nuclei at near barrier energies. Remarkable breakup diversity, i.e., direct and transfer-triggered breakup, has been observed in the reactions induced by ${}^{6,7}$Li and ${}^{9}$Be. Distinct breakup mechanisms are observed for ${}^{6}$Li and ${}^{7}$Li: the ratio of direct breakup (${}^{6}$Li→α+d) is around 70%, almost independent on the interaction energy and target mass; while for ${}^{7}$Li and ${}^{9}$Be, which have higher breakup thresholds than that of ${}^{6}$Li, direct breakup was mainly observed in reactions with high Z targets or low Z targets but with high energies. The small fraction of direct breakup component indicates that the transfer-triggered breakup is the dominant process, which strongly depends on the transfer Q-value and the structure of the target and projectile nuclei. For the case of ${}^{8}$Li, although with the short-lived radioactive nature, it exhibits diverse breakup modes similar to ${}^{6,7}$Li in the measurements with ${}^{209}$Bi. Large yields of unaccompanied α particles were recorded in these reactions, which possibly arises from processes like breakup fragment captured by the target or cluster transfer. For reactions induced by nuclei located close to the drip lines, elastic breakup becomes more important as the breakup threshold decreases. Moreover, it was found that the proton halo nuclei show a lower breakup probability compared to neutron halo nuclei at the same breakup threshold, demonstrating distinct breakup dynamics in proton halo systems.

The reported results clearly indicate that the complete kinematics measurement is the only adequate approach to investigate the breakup mechanisms of exotic nuclear systems at energies close to the Coulomb barrier. However, there is currently a lack of extensive exclusive breakup data, particularly for drip-line nuclei. Owing to the challenges of effective detection of neutrons, detailed measurements of proton drip-line nuclear systems are hence considered as a breakthrough point. To advance our understanding of the breakup dynamics of exotic nuclei, the following points can be considered as areas of focus.

From the experimental perspective, it is necessary to develop new detector arrays with high efficiency to cover the forward angular region in the laboratory frame. CDCC calculations suggest that the elastic breakup angular distributions of ${}^{8}$B+${}^{120}$Sn at near-barrier energies will peak within the angular region around 20${}^{\circ }$. Obtaining a complete understanding of the breakup angular distribution is crucial for refining the structure model of ${}^{8}$B used in the CDCC calculations. Moreover, the information of the Coulomb polarization effect might be revealed as well by investigating the angular correlation between the breakup fragments in the very forward angular region. Therefore, the experimental data at small angles are critical for studying the reaction dynamics induced by a proton nucleus.

From the side of theory, there is a strong need to develop a theoretical framework which can consistently describe various reaction channels. The present theoretical framework cannot deal with reactions have strong coupling effects from both breakup and transfer. Recently, Moschini and Diaz-Torres [66] have proposed an approach of a two-center molecular continuum and the results have demonstrated the usefulness in describing the reactions induced by weakly bound nuclei with a simple dynamical reaction model. This approach could be promising to reveal the dynamical competition among direct reaction processes during the entire collision process.

With the developments of both the experimental and theoretical techniques, we can extend the investigations for the light proton drip-line nuclei, like, ${}^{7}$Be, ${}^{9,10}$C and ${}^{12}$N. These studies will provide valuable opportunities to systematically and comprehensively understand the reaction dynamics of proton-rich nuclei and drive the development of nuclear reaction theory.

## Declaration of competing interest

The authors declare that they have no conflicts of interest in this work.
